# Atrial Fibrillation in COVID-19: Mechanisms, Clinical Impact, and Monitoring Strategies

**DOI:** 10.3390/biomedicines13122889

**Published:** 2025-11-26

**Authors:** Ewelina Młynarska, Katarzyna Hossa, Natalia Krupińska, Hanna Pietruszewska, Aleksandra Przybylak, Kinga Włudyka, Jacek Rysz, Beata Franczyk

**Affiliations:** 1 Department of Nephrocardiology, Medical University of Lodz, Ul. Zeromskiego 113, 90-549 Lodz, Poland; 2 Department of Nephrology, Hypertension and Family Medicine, Medical University of Lodz, Ul. Zeromskiego 113, 90-549 Lodz, Poland

**Keywords:** COVID-19, atrial fibrillation, new-onset atrial fibrillation, telecardiology, heart rate turbulence, transthoracic echocardiography

## Abstract

The coronavirus disease 2019 (COVID-19) pandemic has revealed a close and multifaceted relationship between viral infection, systemic inflammation, and cardiovascular health. Among the cardiac complications of severe acute respiratory syndrome coronavirus 2 (SARS-CoV-2), atrial fibrillation (AF)—especially new-onset atrial fibrillation (NOAF)—has emerged as a major determinant of disease severity and prognosis. Clinical studies and meta-analyses show that 5–10% of hospitalized COVID-19 patients develop AF, with markedly higher rates in critically ill individuals. Both pre-existing and NOAF are independently associated with increased risks of intensive care admission, mechanical ventilation, thromboembolic events, and mortality. The underlying mechanisms involve a combination of cytokine-mediated inflammation, endothelial dysfunction, microvascular injury, and dysregulation of the renin–angiotensin–aldosterone system (RAAS). Viral downregulation of angiotensin-converting enzyme 2 (ACE2) receptors contributes to myocardial fibrosis, while hypoxia, oxidative stress, and autonomic imbalance further promote electrical remodeling and arrhythmogenesis. Post-infectious studies indicate that atrial structural changes and autonomic dysfunction may persist for months, predisposing survivors to recurrent arrhythmias. Technological advances in telecardiology and digital medicine have provided new tools for early detection and long-term monitoring. Wearable electroencephalography (ECG) devices, implantable loop recorders (ILRs), and artificial intelligence (AI)-based diagnostic algorithms enable continuous rhythm surveillance and individualized management, improving outcomes in post-COVID patients. This review summarizes current evidence on the epidemiology, pathophysiology, clinical implications, and monitoring strategies of AF in COVID-19. It underscores the importance of integrating telemedicine and AI-assisted diagnostics into cardiovascular care to mitigate the long-term arrhythmic and systemic consequences of SARS-CoV-2 infection.

## 1. Introduction

The coronavirus disease 2019 (COVID-19) pandemic has profoundly affected global health, exposing intricate interactions between viral infections and cardiovascular health. While the primary clinical focus has often been on respiratory complications, increasing evidence demonstrates that cardiovascular involvement is both common and clinically significant, particularly in patients with pre-existing heart conditions. Among the various cardiovascular complications associated with severe acute respiratory syndrome coronavirus 2 (SARS-CoV-2) infection, new-onset atrial fibrillation (NOAF) has emerged as a major concern. A large multicenter study involving 30,999 hospitalized patients across 120 centers reported that 5.4% developed new-onset atrial fibrillation (NOAF) during hospitalization, with nearly half of these patients dying during their first hospital stay, underscoring the urgent need for effective monitoring and management [1,2]. Similarly, a meta-analysis of over 39 million patients estimated the incidence of NOAF at approximately 10.3% and demonstrated that the presence of atrial fibrillation is associated with an increased risk of mortality [3].

The pathophysiological mechanisms underlying NOAF in COVID-19 patients are multifactorial. Viral-induced inflammatory responses, myocardial injury, and electrolyte disturbances contribute to atrial electrical remodeling, creating a substrate for arrhythmia development [4]. Comorbidities such as arterial hypertension, diabetes mellitus, and pre-existing cardiovascular disease, commonly observed in COVID-19 patients, further increase susceptibility to arrhythmia. Comparative studies have also demonstrated that the incidence of NOAF is higher in COVID-19 patients than in those with pneumonia or acute respiratory distress syndrome (ARDS) unrelated to SARS-CoV-2, suggesting that the virus itself may exert a direct arrhythmogenic effect, independent of pulmonary pathology. Beyond immediate mortality, NOAF has been associated with an elevated risk of major adverse cardiovascular events (MACE), including stroke and heart failure, making early recognition and timely intervention essential to reduce morbidity and mortality in this vulnerable population [5].

The COVID-19 pandemic has also highlighted the need for innovative solutions to monitor and manage cardiovascular conditions remotely, particularly in the context of hospital strain and restricted patient mobility. Telecardiology, a specialized branch of telemedicine, has emerged as a transformative tool in the diagnosis, monitoring, and management of cardiovascular diseases. The origins of telecardiology trace back to the early 1990s, when the first electrocardiograms (ECGs) were transmitted over telephone lines, marking the beginning of remote cardiac care. Since then, rapid advancements in digital communication, wearable devices, mobile health (mHealth) applications, and artificial intelligence (AI) have greatly expanded the potential and scope of telecardiology, establishing it as a critical component of modern cardiovascular medicine [6,7]. 

Telecardiology now encompasses a broad spectrum of applications, including remote patient monitoring, teleconsultations, virtual cardiac rehabilitation, and structured telerehabilitation programs. These technologies enable continuous assessment of vital signs such as blood pressure, heart rate, oxygen saturation, and weight, allowing for early detection of decompensation in chronic conditions like heart failure and arrhythmias. Studies have demonstrated that early identification and intervention through telecardiology can reduce hospitalizations, mortality, and healthcare costs while improving patients’ quality of life, treatment adherence, and engagement in self-management practices. Telemedicine platforms also provide educational resources, lifestyle coaching, and personalized treatment support, empowering patients to actively participate in their care [8].

Technological innovations have further strengthened the impact of telecardiology. Wearable sensors and smart devices provide real-time data, while secure video conferencing facilitates remote consultations and continuous monitoring. In recent studies, ECG recordings and remote cardiac monitoring were performed using devices compatible with the PMcardio application (PM-Cardio, Vilnius, Lithuania), as well as wearable ECG devices used in the TeleCheck-AF project across multiple European centers. Artificial intelligence and machine learning enhance analytical capacity, enabling automated ECG interpretation, predictive risk modeling, and early identification of potentially life-threatening cardiac events, including myocardial infarction and atrial fibrillation. These tools also facilitate collaboration among multidisciplinary healthcare teams, enabling more comprehensive and timely patient care. Despite these advancements, challenges remain, including disparities in access to digital infrastructure, particularly in rural and low-income regions, as well as concerns regarding data security and patient privacy. Addressing these barriers is essential to ensure equitable and sustainable implementation of telecardiology [9,10].

By integrating telecardiology into cardiovascular care models, clinicians can provide timely, patient-centered interventions, optimize chronic disease management, and mitigate the risks associated with viral infections such as COVID-19. This review aims to provide a comprehensive overview of the cardiovascular complications associated with COVID-19, particularly NOAF, while examining the historical development, technological innovations, and clinical applications of telecardiology. Through this synthesis, we aim to inform strategies for improved patient outcomes, enhanced healthcare efficiency, and equitable access to advanced cardiac care in the ongoing pandemic and beyond.

## 2. Epidemiology of AF in Patients with COVID-19

The COVID-19 outbreak in 2019 became a serious global problem as it affected a massive number of people in over 180 countries [11]. According to the World Health Organization, by 17 October 2025, over 770 million people worldwide were affected by SARS-CoV-2, and over 7.1 million deaths were noted. Infection caused by SARS-CoV-2 is mostly associated with pulmonary manifestation; however, based on studies, the cardiovascular system is considered as the most common non-pulmonary manifestation. The level of cardiac biomarkers is increased in 20–30% cases of COVID-19 [12].

At the beginning of the global pandemic, Chinese reports stated that the prevalence of arrhythmias in COVID-19 was 17%, with even higher number in intensive care unit (ICU) patients—up to 44% [13]. Some other investigations from the beginning of the pandemic (2020–2021) showed an occurrence of NOAF around 4–8%. [14,15]. For example, in the study conducted by Bhatla et al. [14], 700 COVID-19 patients were taken into consideration (with a mean age of 50, 45% men, 71% African American, and 11% of them hospitalized in ICU). Among the patients, there were observed 25 AF occurrences, 9 cardiac attacks (all in ICU patients), 9 clinically significant bradyarrhythmias, and 10 non-sustained ventricular tachycardias (NSVTs). It was observed that hospitalization in the ICU was correlated with the prevalence of AF and NSVT [15].

Paris Sara et al. [16] conducted a study in which 696 Italian patients with COVID-19 were taken into consideration from 13 different cardiology centers. Among the patients, 15% (*n* = 106) had a previous history of AF. It was observed that the prevalence of AF was correlated with more in-hospital clinical events such as acute heart failure (25.3% patients with AF vs. 6.3% non-AF patients), multiorgan failure (13.9% patients with AF vs. 5.8% non-AF patients), and especially NOAF (36.8% patients with AF vs. 7.9% non-AF patients). Moreover, an increased risk of death was correlated with the prevalence of AF [16].

In order to estimate possible NOAF in patients with COVID-19, Slipczuk et al. [17] conducted a study investigating a coronary artery calcium (CAC) and epicardial adipose tissue (EAT). The study included 379 patients with the median age 68 years, and males and females were 48.3% and 51.7%, respectively, and 39.3% were Hispanic. Among the comorbidities, the most popular were arterial hypertension—86.8%, hyperlipidemia—70.7%, diabetes—59.9%, asthma or chronic obstructive pulmonary disease (COPD)—49.5%, and coronary artery disease (CAD)—39.8%. The statistical analysis between the groups with and without NOAF showed that there was no significant difference in CHA2DS2-VASc score, age, body mass index (BMI), and incidence of previously listed comorbidities. NOAF was more common in male patients. Patients with NOAF had longer hospitalization, and they needed pressors, intubation, and IV steroids. There was a small difference in potassium level as well (4.7 mEq/l patients with AF vs. 4.4 mEq/l in non-AF patients; *p*  =  0.027). The level of EAT was compared to median volume in the population (92 mL with the norm range of 60–130 mL). It was observed that patients with NOAF had a higher EAT level, and patients who had an EAT level higher than median were in worse condition with a need for intubation or pressors, or deaths were even observed [17].

Marco Zuin et al. [7] conducted a meta-analysis study in which 19 478 173 patients were taken into consideration from five different studies (with a mean age of 56.5 years and where 63% of them were male) in order to estimate the risk of AF incident in COVID-19-recovered patients compared to non-infected patients who developed AF. In this group, there were 5,692,510 patients who recovered from the infection caused by SARS-CoV-2. After a 14.5-month-long follow-up, the pooled occurrence of NOAF was equal to 2.6% of cases compared to non-infected patients in the same period of time. It was concluded that there is a risk of AF incidents in recovered COVID-19 patients [7].

The meta-analysis conducted by Nan-Nan et al. [3] included 80 articles with a total of 39,062,868 patients. Among them, 15 articles were from Asia (6 from Turkey, 4 from China, 2 from India, 2 from Korea, and 1 from Saudi Arabia), 22 studies were performed in North America (all of them from the USA), and 43 studies were performed in Europe (19 from Italy, 7 from Spain, 7 from the UK, 4 from Denmark, 2 from Poland, and 1 article each from France, Portugal, Switzerland, and the Netherlands). Among these investigations, most of them were focused on the occurrence of pre-existing AF (*n* = 74), some were focused on NOAF (*n* = 3), and some of them were focused on both of these clinical situations (*n* = 3). According to the results, the estimated occurrence of pre-existing AF was shown to be equal to 10.5%. However, in this group, it was observed that elderly patients had a 2.5-fold higher prevalence of AF compared to others. Moreover, there were significant differences calculated in the occurrence of AF among different regions—the highest prevalence was shown in European populations, followed by North Americans and Asians. A correlation between the severity of COVID-19 and the occurrence of AF was also observed—the prevalence was about 2.5-fold higher in severe patients [3].

## 3. Pathophysiology and Mechanisms of AF Development in COVID-19

The pathogenesis of AF is complex and multifactorial. Various factors contribute to structural and functional atrial changes that promote its onset and progression. The exact mechanisms remain incompletely understood [18]. AF development is closely associated with both electrical and structural atrial remodeling [19]. Evidence indicates that stretch-induced fibrosis, epicardial adipose tissue, chronic inflammation, autonomic imbalance, and genetic factors play roles [18,20]. Both modifiable and non-modifiable cardiovascular risk factors—including obesity, arterial hypertension, diabetes mellitus, hyperthyroidism, surgery, alcohol use, smoking, and sedentary lifestyle—contribute to AF development [21,22]. AF often first manifests as a paroxysmal arrhythmia, triggered by alcohol, drugs, or inflammation [23].

The underlying pathophysiological mechanisms linking SARS-CoV-2 infection and AF remain incompletely understood [24]. Several mechanisms have been proposed to explain NOAF in COVID-19, including the cytokine storm, downregulation of angiotensin-converting enzyme 2 (ACE2) receptors, endothelial injury, and heightened sympathetic nervous system activation [2,19].

One of the main mechanisms connecting SARS-CoV-2 infection to AF is the induction of systemic inflammation. The infection triggers a strong immune response characterized by the release of proinflammatory cytokines and chemokines, which can affect the heart and cause myocarditis, leading to electrical instability and an increased susceptibility to AF [25,26]. Consistent with this inflammatory response, a study by Stasiak et al. [27] in 2024 involving 51 pediatric patients with pediatric inflammatory multisystem syndrome temporally associated with SARS-CoV-2 (PIMS-TS) demonstrated diverse cardiovascular abnormalities, including pericardial effusion, valvular insufficiency, coronary artery dilatations or aneurysms, and myocardial hypokinesis. AF and COVID-19 share overlapping pathophysiological mechanisms driven by immune-mediated processes, with inflammatory markers such as C-reactive protein (CRP) and interleukin (IL)-6 correlating with disease severity and mortality [19].

Inflammation and reactive oxygen species disrupt the homeostasis of the atrial myocardium, contributing to both the initiation and persistence of AF. In patients with AF, infiltration of inflammatory cells and elevated serum levels of mediators such as tumor necrosis factor-α (TNF-α), IL-1β, IL-6, IL-8, and IL-10 have been observed, correlating with the duration and severity of the arrhythmia [15]. In a 2012 study by Pena et al. [28] involving 17,120 participants, higher CRP levels were significantly associated with an increased risk of incident AF, with each one-unit increase in CRP corresponding to a 28% higher risk after adjustment for confounders. Similarly, in a study by Gundlund et al., 30,304 patients with infection-related AF were compared to 90,912 patients with infection but without AF. During the first year, 36.4% of patients with infection-related AF experienced recurrent AF and 7.6% had thromboembolic events, compared with 1.9% and 4.4%, respectively, in patients without AF. Infection-related AF was associated with a substantially higher long-term risk, suggesting that AF during infection may require management and follow-up similar to non-infection-related AF [23,29].

According to a study by Conen et al. in 2010 [30], systemic inflammation was significantly associated with an increased risk of incident AF in women without prior cardiovascular disease. In this large-scale prospective cohort including 24,734 participants, elevated levels of inflammatory biomarkers such as CRP, soluble intercellular adhesion molecule-1, and fibrinogen were independently correlated with AF incidence. A combined inflammation score integrating these biomarkers demonstrated a strong, graded, and independent relationship between systemic inflammation and AF development during a median follow-up of 14.4 years. In a study conducted by Kallergis in 2007 [31], CRP levels were shown to predict recurrence of AF after electrical cardioversion in patients with persistent AF. Restoration and maintenance of sinus rhythm were associated with a significant decrease in CRP, suggesting that inflammation may be both a cause and a consequence of AF.

Immune activation may further contribute to AF development in COVID-19. Hemodynamic stress and pericyte signaling can trigger myocardial inflammation, with CD4+ T cells playing a role in atrial remodeling. Impaired regulatory T cell function, particularly in patients with chronic inflammatory conditions, may exacerbate microvascular dysfunction and increase AF susceptibility. This bidirectional interplay between AF and immune responses links chronic inflammation to worse COVID-19 outcomes and arrhythmic risk [24].

In severe COVID-19, flow cytometry studies have shown reduced circulating lymphocytes, including CD3+, CD4+, and CD8+ T cells, with lower CD4+ counts linked to worse outcomes. This decrease may result from enhanced CD4+ cell trafficking to inflamed tissues and a shift toward Th1 interferon-gamma-producing cells in the myocardium. Additionally, circulating CD4+ T cells often exhibit exhaustion, a dysfunctional state that can exacerbate inflammation [24].

In a study by Musikantow et al. in 2021 [15], which included 1 420 patients with influenza and 3970 patients hospitalized with confirmed COVID-19, the incidence of AF in COVID-19 patients was comparable to that observed in influenza patients, suggesting that these atrial arrhythmias likely reflect a nonspecific response to severe viral respiratory illness rather than a COVID-19-specific effect.

SARS-CoV-2 may also directly interact with cardiac cells expressing ACE2 receptors [24]. A study by Chen et al. in 2020 [32] demonstrated that ACE2 is highly expressed in pericytes of adult human hearts, indicating a particular vulnerability of the myocardium to SARS-CoV-2 infection. Patients with pre-existing heart failure exhibit even higher ACE2 expression, which may predispose them to cardiac complications and more severe disease progression following infection. Pericytes, which envelop endothelial cells and maintain microvascular stability, play a crucial role in vascular homeostasis. Their injury during infection can disrupt endothelial–pericyte interactions, leading to inflammation, fibrosis, thrombosis, and microvascular leakage [24].

Endothelial damage and pericyte activation in COVID-19 stimulate the release of growth factors such as vascular endothelial growth factor (VEGF), angiopoietin-2 (Ang2), and transforming growth factor beta 1 (TGF-β1), all of which promote inflammation, fibrosis, and electrophysiological alterations conducive to atrial fibrillation development. Increased hydrostatic pressure further shortens action potential duration and enhances AF susceptibility via activation of the renin–angiotensin–aldosterone system (RAAS) [24].

ACE2 normally converts angiotensin I and II into Ang(1–9) and Ang(1–7), peptides with protective effects that counteract the classical RAAS pathway. However, when SARS-CoV-2 binds to ACE2 for cellular entry, the receptor is downregulated, impairing angiotensin II (ATII) degradation and tipping the balance toward ATII dominance. This shift promotes cardiac hypertrophy, vasoconstriction, oxidative stress, and fibrosis. Furthermore, ACE2 catabolizes TGF-β1, and its reduced activity during infection may contribute to atrial remodeling and increased vulnerability to AF in COVID-19 patients [19].

CD147 may facilitate SARS-CoV-2 entry into cardiomyocytes and promote AF by upregulating cytokine expression, inducing oxidative stress, and activating IL-18, which, in turn, stimulates matrix metalloproteinases (MMPs) such as MMP-9. Elevated MMP-9 levels contribute to extracellular matrix degradation, TGF-β1 activation, and adverse myocardial remodeling, and they are also observed in COVID-19 patients. Additionally, viral spike proteins binding to sialic acid, particularly N-acetylneuraminic acid, may activate Ras homolog family member A (RhoA) signaling, promoting cardiac fibrosis and atrial enlargement, further contributing to AF pathophysiology [19].

In severe cases of COVID-19, patients frequently experience marked hypoxia, a significant reduction in blood oxygen levels. Sustained hypoxia can adversely affect cardiac function, leading to structural and electrical remodeling of the heart and increasing the risk of arrhythmias, including new-onset AF [26]. Hypoxia plays a critical role in both the initiation and maintenance of AF. Hypoxia-inducible factor (HIF), the master regulator of cellular oxygen homeostasis, modulates multiple chemokines and cytokines involved in structural remodeling, such as fibrosis, and can also contribute to electrical remodeling of the atria [33].

According to a study by Gramley et al. in 2010 [34], AF is closely associated with increased expression of hypoxia- and angiogenesis-related markers in atrial tissue. The authors observed significant progression of atrial fibrosis in AF patients compared to those in sinus rhythm, accompanied by elevated cytoplasmic levels of hypoxia-inducible factors (HIF1α and HIF2α), VEGF, and its kinase insert domain receptor (KDR). These findings suggest that hypoxia and angiogenic signaling may play a role in atrial remodeling, potentially contributing to the initiation and maintenance of AF.

In COVID-19, as in other viral infections, sympathetic nervous system activation occurs. The enhanced sympathetic tone may contribute to AF by increasing Ca2^+^ influx and overload in cardiomyocytes, which promotes spontaneous diastolic Ca2^+^ release through ryanodine receptors (RyR). This, in turn, generates delayed afterdepolarizations and action potentials, thereby raising the likelihood of AF episodes [19,26].

Mitochondrial dysfunction, characterized by nicotinamide adenine dinucleotide (NAD^+^) depletion, impaired Ca^2+^ handling, and disrupted microtubule networks, reduces adenosine triphosphate (ATP) production and promotes oxidative stress, creating a proarrhythmic substrate that may link COVID-19-induced cellular stress to the development of atrial fibrillation [35,36].

Finally, emotional and psychological stress linked to SARS-CoV-2 infection can indirectly impact cardiac function. Stress and anxiety may activate the sympathetic nervous system, increasing atrial excitability and potentially triggering AF in predisposed individuals [26]. In a study conducted in the South Sinai Governorate of Egypt involving 382 COVID-19 patients, 91.6% experienced symptoms of depression, anxiety, and stress within six months after infection. Severe or extremely severe anxiety was reported in 89.6% of participants, depression in 69.1%, and stress in 58.1%. Hospital admission was identified as the strongest predictor of these psychiatric disorders [37]. Similarly, in a meta-analysis and systematic review conducted by Wu et al. in 2022, including 13 studies with 5,329,908 participants, adverse psychological factors were found to significantly increase the risk of AF, by 10% for anxiety, 15% for anger, 25% for depression, and 18% for work-related stress [38].

## 4. Clinical Significance and Prognosis

AF has emerged as a clinically relevant cardiac manifestation in patients with COVID-19, representing a frequent complication during infection and hospitalization. Approximately one in five patients with COVID-19 have a prior history of AF, whereas NOAF commonly develops during hospitalization. Clinical evidence indicates that AF, whether pre-existing or newly developed during COVID-19, is associated with adverse in-hospital outcomes and may predispose to recurrent or persistent arrhythmic events beyond the acute phase [19].

Accumulating clinical data indicate that AF in patients with COVID-19 correlates with a more severe clinical course. Large cohorts and meta-analyses consistently link AF, particularly NOAF, to higher rates of critical illness and respiratory failure [39,40]. A recent meta-analysis of critically ill COVID-19 patients admitted to the ICU demonstrated that NOAF was significantly associated with higher risks of in-hospital and ICU mortality, as well as increased incidence of complications, including mechanical ventilation, acute myocardial infarction (AMI), acute kidney injury (AKI), and the need for renal replacement therapy (RRT). In contrast, the risk of pulmonary embolism was not significantly different between groups. Overall, NOAF in severe COVID-19 appears to reflect systemic illness and multiorgan dysfunction rather than isolated cardiac pathology [39].

In a retrospective cohort of 647 hospitalized COVID-19 patients, new-onset atrial arrhythmias occurred in 6.3% (NOAF 5.4%, atrial flutter (AFL) 0.9%). Older age (≥65 years) and sepsis were identified as independent predictors of NOAF/AFL [40]. Several large studies have reported that AF is associated with higher mortality and adverse clinical outcomes. In a multicenter retrospective cohort of over 23,000 hospitalized COVID-19 patients, NOAF occurred in approximately 5% and was independently associated with adjusted in-hospital and 30-day mortality rates of 16.5% and 22.7%, respectively, reflecting nearly a twofold higher risk of death compared with patients without or pre-existing AF (adjusted odds ratio (aOR) 2.24; 95% CI 1.93–2.60) [41].

Consistent observations were also reported in the large multicenter COVID-PREDICT study, which included more than 3000 hospitalized patients. In this cohort, NOAF occurred in 5.4% of patients and was significantly associated with a higher risk of death (aOR 1.71; 95% CI 1.17–2.59), ICU admission (aOR 5.45; 95% CI 3.90–7.61), and complications such as ARDS and thromboembolic events, which occurred more frequently in patients with NOAF [42]. Although this association remained statistically significant after adjustment, it is believed to reflect the underlying severity and systemic prothrombotic state of COVID-19 rather than a direct causal effect of AF itself [1,42,43]. By contrast, pre-existing AF showed no independent effect on survival, and its associations with ICU admission and complications were less pronounced than for NOAF [42]. Overall, these findings suggest that NOAF is more frequently observed in patients with severe systemic illness and correlates with poorer clinical outcomes [42]. A summary of the major clinical studies evaluating the prognostic impact of AF in COVID-19 is presented in Table 1.

Further evidence comes from a multicenter cohort analysis, in which patients with AF/AFL had significantly higher peak levels of inflammatory and myocardial injury markers, including CRP, cardiac troponin I, B-type natriuretic peptide (BNP), ferritin and D-dimer, compared with those without AF/AFL. In this cohort, atrial arrhythmias were closely associated with elevated markers of systemic inflammation and cardiac injury and were independently linked to an almost twofold increase in 30-day mortality, with newly detected AF/AFL conferring the highest risk [44].

Additionally, a pooled analysis including 80 studies and more than 39 million COVID-19 patients assessed the prevalence of AF and its association with mortality. The prevalence of pre-existing and NOAF was approximately 10% each, with markedly higher rates among elderly individuals and those with severe disease. Severe COVID-19 cases exhibited around a 2.5-fold higher incidence of AF compared with non-severe cases. Both pre-existing and NOAF were independently and significantly associated with increased mortality, suggesting a potential prognostic role of AF in COVID-19, particularly for NOAF. Subgroup analysis showed the highest mortality risk among Asian cohorts, although this difference did not reach statistical significance [3].

Registry data indicate, however, that this observed association may in part reflect confounding by disease severity. In a multicenter analysis of over 30,000 hospitalized patients from the American Heart Association COVID-19 Cardiovascular Registry, NOAF occurred in 5.4% of cases. These patients exhibited markedly higher in-hospital mortality (45.2% vs. 11.9%) and major adverse cardiovascular events (MACE; 23.8% vs. 6.5%) than those without AF. After multivariable adjustment for comorbidities and illness severity, the excess mortality risk was no longer significant (adjusted hazard ratio (aHR) 1.10; 95% CI 0.99–1.23), while the association with MACE remained but was attenuated (aHR 1.31; 95% CI 1.14–1.50) [1].

Additional data from a cohort of patients with severe COVID-19 further suggest that not all AF phenotypes carry the same prognostic weight. Persistent and permanent AF were associated with the highest mortality risk and represented independent predictors of death in multivariable analysis, whereas paroxysmal AF carried a comparatively lower risk. These findings suggest that AF burden and the chronic atrial substrate may modulate prognosis in patients with severe COVID-19 [45].

**Table 1:** Prognostic impact of atrial fibrillation in COVID-19: overview of major clinical studies.

Study/Year	Sample Size (N)	Type of AF (Atrial Fibrillation)	Incidence/Prevalence	Main Clinical Outcomes	Adjusted Association	Interpretation
ICU meta-analysis [39]	ICU cohort	NOAF	~5–6%	↑ICU/in-hospital mortality; ↑AMI, AKI, RRT; PE ns	Yes	NOAF reflects multiorgan failure
Retrospective cohort [40]	647	NOAF/AFL	6.3%	Older age and sepsis = strongest predictors	Yes	Arrhythmia driven by systemic illness/sepsis burden
Multicenter study [41]	>23,000	NOAF	~5%	↑30-day mortality	aOR~2.24	Independent predictor of short-term mortality
COVID-PREDICT [42]	3064	NOAF	5.4%	↑ICU admission, ARDS, thromboembolism	aOR 1.71	NOAF tracks severity rather than primary cardiac cause
AHA Registry [1]	>30,000	NOAF	5.4%	Crude mortality ↑ but attenuated after adjustment	aHR 1.10 (ns)	Mortality signal largely confounded by illness severity
Pooled meta-analysis [3]	>39,000, 000	Pre-existing AF + NOAF	~10% each	↑Mortality in both AF phenotypes	Yes	AF functions as a global prognostic marker
VA cohort (long-term) [46]	150,000 vs. 11,000,000	Post-acute AF	persists ≥ 12 months	Long-term AF susceptibility	aHR 1.71	Persistent vulnerability beyond acute phase

Abbreviations: AF—atrial fibrillation; NOAF—new-onset atrial fibrillation; AFL—atrial flutter; ICU—intensive care unit; ARDS—acute respiratory distress syndrome; TE—thromboembolic events; PE—pulmonary embolism; AMI—acute myocardial infarction; AKI—acute kidney injury; RRT—renal replacement therapy; aOR—adjusted odds ratio; aHR—adjusted hazard ratio; ns—non-significant; ↑—increased risk/higher incidence.

While NOAF is thought to primarily reflect acute systemic illness during hospitalization, AF also appears to have relevance beyond discharge, with emerging evidence indicating an increased risk of incident AF in the post-acute phase. In a large nationwide cohort of more than 150,000 survivors compared with over 11 million controls, AF risk remained elevated for at least 12 months (aHR 1.71; 95% CI 1.64–1.79). This excess was present even among non-hospitalized cases and rose with greater acute illness severity. It was also observed in individuals without prior cardiovascular disease, suggesting a longer-term post-infectious susceptibility to arrhythmia [46].

Mechanistic insights further support this vulnerability. In a Holter-based study, atrial heart rate turbulence (HRT), an index of autonomic baroreflex function derived from short-term fluctuations in sinus cycle length after supraventricular premature beats, was assessed using turbulence onset (early acceleration) and turbulence slope (subsequent recovery). Individuals who had recovered from severe COVID-19 demonstrated significantly more frequent abnormal HRT onset compared with both controls and those with mild or moderate disease. The impairment correlated with chest computed tomography (CT) severity but not with time since infection or the number of previous PCR-confirmed episodes. Severe disease, hypertension, and smoking remained independent predictors of abnormal atrial HRT, indicating that autonomic dysfunction after severe COVID-19 may contribute to an elevated long-term risk of AF. These findings are consistent with early autonomic dysfunction that may be relevant to later AF risk [47].

Beyond the development of new-onset AF, observational data also show that a history of AF itself confers a worse long-term prognosis in COVID-19 survivors, particularly with respect to all-cause mortality. In a large nationwide cohort, patients with pre-existing AF had a significantly higher short- and long-term mortality risk following hospitalization for COVID-19, even after adjustment for age and comorbidities. This indicates that AF may identify a subgroup of patients with persistently elevated post-discharge risk that extends beyond the acute infectious phase [48].

Clinical data further support this interpretation: AF during COVID-19 occurs predominantly in patients experiencing the greatest systemic and cardiopulmonary stress, and in observational cohorts it closely parallels illness severity and multiorgan dysfunction, suggesting that AF reflects a global rather than isolated atrial process [14].

Taken together, these data suggest that AF in the context of COVID-19 functions primarily as a marker of systemic and autonomic vulnerability rather than an isolated arrhythmic event. Its presence identifies patients in whom the inflammatory and multi-organ burden of COVID-19 is greater and more sustained and who may therefore benefit from closer post-discharge clinical follow-up, particularly when additional risk factors are present [14,48].

## 5. Treatment and Management Strategies

AF is associated with an increased risk of major cardiovascular events, including ischemic stroke or systemic embolism, heart failure, hospitalizations, reduced quality of life, and higher mortality. It is a complex condition requiring comprehensive, multidisciplinary, and long-term management, posing a substantial burden for patients and healthcare systems [49]. AF is not only linked to chronic cardiovascular morbidity but may also complicate acute illnesses such as COVID-19. In patients with SARS-CoV-2 infection, AF has been associated with worse clinical outcomes, including higher mortality, increased risk of heart failure, and prolonged hospitalizations [50]. Patients with AF are more likely to be hospitalized due to COVID-19 and to receive antiviral therapy. Additionally, up to 10% of individuals with acute respiratory distress syndrome develop NOAF. AF has been reported in approximately one in five patients hospitalized with COVID-19, with higher prevalence in severe cases [51].

While arrhythmia-directed interventions are largely investigational in this context, understanding AF’s prognostic significance can help refine risk stratification and guide therapy, particularly when healthcare resources are constrained. In critically ill COVID-19 patients with AF, management typically involves rate control combined with anticoagulation. Procedures such as transesophageal echocardiography (TEE) or cardioversion are challenging in critically ill individuals and are further complicated by the risk of viral exposure to healthcare personnel [50].

AF patients may be asymptomatic or experience symptoms at normal, high, or low ventricular rates. When choosing between rhythm control (restoring and maintaining sinus rhythm) and rate control (regulating ventricular rate while allowing AF to persist), the main goal is symptom relief and maintaining appropriate ventricular rates [52,53,54]. In a 2002 study by Van Gelder et al. [53] involving 522 patients with persistent AF after prior cardioversion, rate control was non-inferior to rhythm control in preventing cardiovascular death and related complications. After a mean follow-up of 2.3 years, sinus rhythm was maintained in 39% of the rhythm control group versus 10% of the rate control group. The composite endpoint—including cardiovascular death, heart failure, thromboembolism, bleeding, pacemaker implantation, and serious drug side-effects—occurred slightly less often with rate control (17.2% vs. 22.6%), supporting it as a reasonable option for recurrent persistent AF. A meta-analysis by Chatterjee et al. similarly found no significant difference between rate and rhythm control regarding mortality, stroke, or heart failure. Rate control reduced rehospitalizations, while rhythm control showed a potential mortality benefit in patients under 65 years [54]. Evidence on the effectiveness of rhythm and rate control strategies in patients with AF and concurrent COVID-19 infection remains limited [10].

According to the 2023 ACC/AHA/ACCP/HRS Guideline for the Diagnosis and Management of AF, shared decision-making is recommended to weigh rhythm versus rate control strategies, considering clinical presentation, comorbidities, medications, and patient preferences. In suitable patients without heart failure, rate control aims for individualized targets based on symptoms; a resting heart rate <100–110 bpm is generally reasonable [55]. Rhythm control can be achieved using antiarrhythmic drugs (AADs; classes Ia, Ic, or III), cardioversion, or AF ablation. Beta-blockers are first-line agents for rate control, with calcium channel blockers and digoxin as alternatives or adjuncts [56].

Electrical cardioversion is rapid and effective, particularly in hemodynamically unstable patients, and its success can be enhanced by pretreatment with AADs, energy optimization, and proper electrode positioning [55]. Catheter ablation is an effective rhythm control option for symptomatic patients, especially when AADs are ineffective, contraindicated, or not preferred. In selected younger patients with few comorbidities, ablation may be considered first-line to improve symptoms and prevent AF progression. Ablation is also useful in patients with symptomatic atrial flutter or during procedures for other supraventricular arrhythmias [55]. In the context of COVID-19 infection, chemical cardioversion is preferred over electrical cardioversion as an initial treatment for hemodynamically stable patients [57]. Hypoxemia, inflammation, and electrolyte imbalances (hypokalemia, hypomagnesemia, acidosis) can trigger AF in COVID-19 and should be corrected; persistent AF may require AAD therapy [5].

Urgent cardioversion should be considered in hemodynamically unstable patients, including those with acute myocardial infarction or heart failure, when new-onset AF contributes to instability. In critically ill patients with AF-related instability, intravenous amiodarone is the antiarrhythmic of choice but should be used cautiously due to pulmonary toxicity and hypotension risks. Intravenous diltiazem may provide adequate rate control in selected cases. Transthoracic echocardiography (TTE) is recommended when cardiac dysfunction or hemodynamic instability is suspected, and early anticoagulation can often replace TEE. Cardiac computed tomography may serve as an alternative to evaluate left-atrial appendage thrombus before cardioversion in stable patients [10].

In hospitalized patients receiving antiviral therapy who develop new-onset or recurrent AF without hemodynamic instability, discontinuation of AADs and initiation of rate control therapy—preferably with beta-blockers or, if not contraindicated, non-dihydropyridine calcium channel blockers, with or without digoxin—is recommended to enable safe antiviral treatment and reduce QT prolongation risk. QT prolongation risk may be further increased by concomitant use of QT-prolonging agents (e.g., hydroxychloroquine, azithromycin, lopinavir/ritonavir), myocardial inflammation, and electrolyte disturbances [10]. Therefore, potential drug–drug interactions between antivirals and AADs should always be assessed before therapy [10,51].

Amiodarone (class III AAD) is metabolized by CYP3A4 and CYP2C8, and inhibitors of these enzymes can increase plasma levels [58]. Guidelines recommend caution with co-administration due to amiodarone’s long half-life [47]. Nirmatrelvir–ritonavir (Paxlovid), a protease inhibitor with strong CYP3A4 inhibition, can elevate amiodarone levels and associated adverse effects [59]. Propafenone (class Ic AAD), metabolized by CYP2D6, CYP3A4, and CYP1A2, may also interact with enzyme inhibitors, increasing the risk of QRS prolongation, ventricular arrhythmias, and interactions with beta-blockers [50]. Close monitoring and dose adjustment are advised, with ECG surveillance for QTc prolongation or torsades de pointes. Overall, AADs exhibit mild interactions with tocilizumab, moderate with corticosteroids, and moderate-to-severe with antibiotics, while no interactions have been reported with remdesivir [51].

Verapamil and diltiazem inhibit CYP3A4 and P-glycoprotein; co-administration with inhibitors may necessitate dose reduction. Beta-blockers (metoprolol, carvedilol, propranolol) are metabolized via CYP2D6; digoxin is eliminated via P-glycoprotein, requiring monitoring. No dose adjustment is needed with tocilizumab. Based on clinical experience and drug–drug interaction risk, beta-blockers are first-line for rate control [51].

Initial observations in Wuhan, China, followed by worldwide reports, suggested that thromboembolic complication incidence ranged from 15% to 85% [58]. Current guidelines recommend anticoagulation for patients with AF and CHA2DS2-VASc score ≥2 in men (≥3 in women), and it should be considered for those with scores ≥1 in men (≥2 in women) [55]. Hospitalized COVID-19 patients are typically over 65 years old and present with two or more comorbidities in approximately 70% of cases, so most AF patients require long-term anticoagulation [60,61].

In hemodynamically stable hospitalized COVID-19 patients with AF, anticoagulation may be achieved using unfractionated heparin (UFH), low-molecular-weight heparin (LMWH), or direct oral anticoagulants (DOACs), depending on oral intake, renal function, and clinical status. Some investigational COVID-19 therapies, such as lopinavir/ritonavir (CYP3A4) and antimalarials (P-glycoprotein), may interact with DOACs, increasing bleeding risk; in such cases, DOACs should be avoided. DOACs are generally preferred over vitamin K antagonists (VKAs) due to fixed dosing and better safety profiles [10].

According to Tomaszuk-Kazberuk et al., AF patients receiving VKA therapy hospitalized for COVID-19 pneumonia—including those with prosthetic valves or moderate-to-severe mitral stenosis—should be transitioned to LMWH or UFH, primarily due to drug–drug interaction risk [51]. VKA therapy requires frequent international normalized ratio (INR) monitoring, increasing infection risk, so VKAs are reserved for patients with mechanical valves or antiphospholipid syndrome [10]. Vitamin K deficiency, affecting proteins C, S, and matrix Gla protein, may worsen COVID-19 outcomes; Dutch data show low vitamin K is associated with higher ventilation needs and mortality [62].

COVID-19 treatment has evolved significantly. Experimental therapies including azithromycin, chloroquine, hydroxychloroquine, convalescent plasma, interferon beta, and lopinavir/ritonavir have proven ineffective, and some interact with anticoagulants or AADs. Current management of mild cases focuses on rehydration, antipyretics, antitussives, and inhaled budesonide. Moderate-to-severe pneumonia is treated with remdesivir, tocilizumab, dexamethasone, or methylprednisolone. Most hospitalized patients require respiratory support, from supplemental oxygen to mechanical ventilation or extracorporeal membrane oxygenation (ECMO). Antibiotics are reserved for confirmed or suspected bacterial co-infections [51].

A 2022 study by Bistrovic et al. [63] in 1752 hospitalized COVID-19 patients (876 treated with remdesivir, 876 matched controls) found that 188 patients had AF. AF patients had higher mortality than non-AF patients (50.5% vs. 29.2%). Remdesivir improved survival (29.2% vs. 33.8%); among AF patients, mortality was lower with remdesivir compared to controls (43.2% vs. 57%), particularly when administered before high-flow oxygen therapy or mechanical ventilation. No benefit was observed when given during advanced respiratory support. Early remdesivir may improve survival in COVID-19 patients with and without AF, possibly via antiviral effects and better heart rate control.

The COVID-19 pandemic has profoundly impacted medicine. Despite telemedicine and hospital protocols, access to care remains limited, especially for chronic patients, risking exacerbations and complications. Clinicians must remain vigilant for COVID-19′s early and late effects, its impact on chronic diseases, and potential therapy interactions [51]. During the pandemic, in-person visits were often replaced by video consultations, and medical information was provided digitally to ensure safe care. Hospitals increasingly adopted telemedicine and remote monitoring tools, including personal ECG devices. However, many countries still lack regulatory frameworks for authorization and reimbursement, and the efficacy and safety of these solutions, particularly in emergencies, remain uncertain. Further research is needed before telemedicine can be fully implemented in routine AF management [64].

## 6. Monitoring and Future Directions

The key issue after suffering COVID-19 is that patients, especially those with heart rate disorders such as AF, should be constantly monitored because of possible unpredictable process and outcomes of this supraventricular arrhythmia, like ischemic stroke or heart failure [65,66]. In the research from 2022 by Dewland et al. [67], 51 adult patients with no significant medical history were submitted to 14-day continuous ambulatory electrocardiographic monitoring after a COVID-19 diagnosis (median time between the viral test and the examination was 75 days). In the study by Khan et al. [50], it is shown that temporary transient rhythm disturbances referred to almost all patients. Most of the situations were associated with sinus rhythm and sinus tachycardia. This means that although atrial fibrillation is probably not one of the most common short-term consequences of COVID-19 infection, other heart rhythm disorders may appear. Moreover, there is a need for discovering long-term side-effects of this viral disease, and monitoring can help in reaching this goal.

The study by Hamdy et al. [68] from 2024 is concentrated basically on detecting ventricular arrhythmias, but it emphasizes the clinical rationale to prevent them from more serious consequences. A group of 60 people with no earlier cardiac disorders who were between one and six months after recovering from COVID-19 and underwent ventricular arrhythmias during the infection were submitted to 24 h ambulatory Holter ECG and TTE. Scientists propounded a conclusion that this group of patients frequently suffer from prolonged systemic inflammation with increased CRP and troponins as well as subclinical myocarditis and impairment of left ventricle function. These parameters may predispose to development of hypertension, heart failure, enlargement of left atrium, and, finally, atrial fibrillation.

COVID-19 infection can trigger arrythmias probably because of cytotoxic injury and microvascular damage, which cause increased fibroblast proliferation, reduced myocardial compliance, receptor and mediator dysfunction, and gap junction disorders. This leads to uncontrolled propagation of electric impulses within the myocardium [69]. Heart structure remodeling is a long-lasting issue, so patients should be observed for many months, which raises the chance of noticing expanding complications.

Unfavorable structural changes of the left atrium were also mentioned in a study from 2023 by ZeinElabdeen et al. [70]. Usage of speckle tracking echocardiography (STE) in 63 COVID-19 patients with no significant past medical history 4–12 weeks after recovery shows that nearly one-third of examined people experienced breathlessness and deterioration of exercise tolerance connected with impaired left-atrial strain, which can contribute to disorder of left-ventricular diastolic function.

A useful option in non-invasive monitoring is wearable sensors [71]. Participants downloaded the application ‘MyDataHelps’ to their smartphones. The measured parameters were step count, resting heart rate, and sleep quantity. They were also encouraged to complete a survey about their symptoms in case of COVID-19 infection or vaccination. In this study above, it is illustrated that there is a significant difference in results between groups of people with and without long-COVID; moreover, the disease process was more severe in women, younger people, and unvaccinated patients. The data gathered by the sensors help doctors observe the well-being of their patients and help patients cope with long-term consequences of the viral disease.

There are also studies based on observations of heart rate variability (HRV) [72]. These are subtle (millisecond-long) differences in periods between two heart beats dependent on organism’s adaptability to different situations and on the balance between work of the sympathetic and parasympathetic nervous systems. Greater fluctuations in this indicator suggest more effective functioning of the autonomic nervous system. Among factors that have an impact on HRV are age, gender, alcohol, smoking, circadian rhythm, elements, or the work environment, such as electromagnetic devices, as well as infections. Measuring HRV during COVID-19 infection can be valuable because systemic inflammation caused by the disease has an influence on the parasympathetic nervous system’s work. Natarajan et al. [73] conducted their examination using a Fitbit and photoplethysmogram (PPG) and noticed that, in most cases, HRV decreases after a positive reverse-transcriptase polymerase chain reaction (RT-PCR) COVID-19 test. Similar results appeared in the study organized by Hasty et al. [74]. The applied technologies were Warfighter Monitor and ECG. Observed decreases in HRV correlated with elevation of CRP level during the infection. Another article’s authors deal with continuous monitoring of pregnant women with COVID-19 diagnosis [75]. Participants wore Ava bracelets. There was also usage of PPG. Apart from changes in HRV, it was noticed that resting heart rate (RHR) and respiratory rate (RR) increased when HRV reached lower values. Additionally, duration of deep sleep was reduced. There were also trials to predict onset of COVID-19 disease based on measured HRV level. Hijazi et al. present an AI model that consisted of two blocks that were helpful in differentiation in terms of whether a person is probably SARS-CoV-2-affected or not [76]. The first part of this system is focused on measuring HRV and beats per minute (BPM), and the second block is responsible for very accurate sequential data processing (for example, time series data). Following the scientists’ conclusions, these algorithms offer a possibility to suspect developing COVID-19 around 2 days before symptom exposure. An analogous study was conducted by Hirten et al. [77]. They described an association between data gathered with the aim of Apple Watches worn by patients (HRV) and the onset of viral infection. Noticeable changes in this parameter took place even 7 days before initial symptoms.

Artificial intelligence algorithms were also a part of remote monitoring of ECG in a group of patients using a biosensor—S-Patch EX [78]. The idea of this project was to help general practitioners make appropriate treatment decisions in case the patients suffered from cardiovascular disorders as a result of COVID-19 infection. In a group of 40 participants, almost one-third (13 patients) experienced arrhythmias, such as atrial fibrillation, supraventricular tachycardia, or ventricular tachycardia. In comparison, standard monitoring led to detecting abnormalities only in 7 people out of 200—but, finally, more of them (25) had to visit a medical care institution because of dyspnea, palpitations, or chest pain.

There was also a comparison between usage of a 72 h wearable ECG patch and 24 h Holter ECG monitoring in cardiogenic vertigo detection (health discomfort connected mostly to bradyarrhythmias) [79]. The results of the study showed that the effectiveness of these methods of patient remote monitoring is similar, although more patients preferred to wear patches for their convenience. Other important methods necessary in preventing serious consequences of arrhythmias are Mobile Cardiac Outpatient Telemetry (MCOT; externally located monitor registering heart electrical activity) and Implantable Loop Recorder (ILR; ECG-measuring device implanted subcutaneously) [80]. They are mostly used to detect atrial fibrillation in patients after incident of ischemic stroke to avoid similar events. The authors of the study demonstrate that both of them, especially MCOT, contribute to a decrease in patient readmissions to hospital and to higher survivability. The conclusion is that for detecting intermittent (including asymptomatic) periods of AF, the best options are ECG patches or ECG Holter recorders worn for 72 h–14 days, whereas smartwatches (PPG/PPG + ECG) can play a significant role in population screening and continuous oversight over convalescents. On the other hand, ILR could be successfully used in long-term control (years). A general summary of the mentioned methods of patient monitoring is presented below in Table 2.

According to these issues, there is an undeniable need for leading long-held registries of patients’ post-COVID-19 health implications. This will contribute not only to deepen scientists’ knowledge but also to effective development of medical care services offered to convalescents. The key issue is to encourage patients to report their symptoms systematically and cooperate with their doctors [81]. These solutions can be priceless in cases of another pandemic occurrence [82]. Górska et al. mention noting variable categories (before, during, and after infection) like demographics and lifestyle habits, genomics, quality of life, comorbidities, vaccination, exposure, physical examination, presentation, vital signs, laboratory tests, imaging, hospitalization, complications, treatment, immune response, and functional tests [83].

It is important to diversify sources of information—surveys, clinical data, official registries, electronic health records (EHRs), images, or biomarkers. In the PARTMO study (mentioned above) led by Chow et al. [78], a virtual model was proposed that can be useful in primary healthcare to telemonitor COVID-19 convalescents—even young, asymptomatic people with no significant medical history—in terms of potential cardiovascular repercussions, especially arrhythmias, with the aim of a wearable S-Patch biosensor.

Moreover, scientists introduced an application called PMcardio AI, which specializes in detecting episodes of AF in hospitalized patients with COVID-19, reaching the effectiveness of an experienced cardiologist and outdoing infectious disease specialists. The diagnostic accuracy was 100% in an examined group of 116 patients [9].

There were also other trials that make use of gathered information and create AI algorithms that will bond and interpret information, supporting general practitioners. Hidden Markov Models (HMMs) are tools which define a set of hidden conditions and judge the probability of changes between them and the probability of appearance of expected observations [84]. They can be based on different data such as ECG, ambulatory blood pressure (ABP), or PPG. There are also models consisting of neural network structures [85]. A Convolutional Neural Network (CNN) is a type of one-directional neural network that learns thanks to filter optimization. They are used in data processing (including, for example, text, image, and sound) and creating prognoses. Then, Recurrent Neural Networks (RNNs) focus on signal feedback—stimulation of one element can trigger the generation of a lot of new sequences of features. A Deep Convolutional Neural Network (DCNN) is a kind of CNN with multiple layers. Long Short-Term Memory (LSTM) is a variation of RNN that is effective in information selection. It controls which data should be saved and which should be deleted from memory. A similar solution is a Gated Recurrent Unit (GRU), which uses gating in managing the flow of information. It reduces the problem of ignoring some data from the past. Authors of a study comparing these models noticed that matching of an eight-layer CNN with a short-cut connection and one-layer LSTM (8CSL) is not only especially efficient in capturing demanded features but also in finding long-term associations between pieces of information.

There is no clear-cut evidence that vaccination against SARS-CoV-2 has a direct impact on reducing risk of atrial fibrillation as a consequence of COVID-19 infection, but it is safe to say that it is definitely worth receiving such a vaccine. It gives the possibility of avoiding serious implications of the disease because it helps the human organism prepare for quick reaction and to eradicate virions thanks to the entered specific antibodies. 

Nevertheless, there were some investigations into whether vaccines contribute to the development of arrhythmias. There was a study by Shi et al. [86] analyzing the frequency of arrhythmias in COVID-19-vaccinated people and in non-COVID19-vaccinated people. Rhythm disorders were more common in the first group (3790.4 cases per million doses versus 13.7 cases per million doses). The overall prevalence of arrhythmia after receiving a vaccine was 561.3 cases per million doses. The research of Deshmukh et al. [87] revealed a slight increase in AF incidence during 3 months after first-dose vaccination in a group of 7757 people with cardiac implantable electronic devices (CIEDs), but this may also be a result of coexistent age-progressive cardiac disorders. The authors of a summary from 2023 [88] indicate that the most common cardiac adverse effects of COVID-19 messenger ribonucleic acid (mRNA) vaccines are myocarditis and pericarditis. Besides them, some cases of arrhythmias were reported after Pfizer-BioNTech vaccine that were predominantly premature ventricular contraction, non-sustained polymorphic ventricular tachycardia, sustained unstable ventricular tachycardia, torsade de pointes, ventricular fibrillation, transient but recurrent complete heart block, and extrasystoles, whereas Moderna vaccine was associated mostly with ventricular tachycardia storm in long QT. There are some molecular mechanisms suspected of provoking these disorders. One of them is that spike protein of SARS-CoV-2 competes for ACE2 with ATII, and then increased accumulation of ATII contributes to the development of overall inflammation, thrombosis, and endothelial damage. Moreover, ATII leads to changes in transmembrane conductance and fosters generation of reentrant rhythms which destabilize the heart’s synchronized work. The authors of the study of Kattubadi et al. [89] also paid attention to side-effects of vaccines from several producers. The most cases of atrial fibrillation were connected with Pfizer (2.6%), followed by Moderna (1.8%) and Astra Zeneca (0.6%), in comparison with influenza vaccine (0.4%). No correlation was observed between Johnson & Johnson’s vaccine and incidents of AF.

Ischemic stroke, as well as pulmonary embolism (PE), belong to the most serious consequences of deep vein thrombosis (DVT). According to the study by Kim et al. [90], patients with COVID-19, regardless of any heart rhythm disorders, have an increased risk of DVT and PE, which indicates the challenging role of anticoagulation management to avoid these cardiovascular complications. The trial conducted by Lemos et al. [91] demonstrates that administration of enoxaparin in therapeutic doses leads to an increase in the partial pressure of oxygen/fraction of inspired oxygen (PaO_2_/FiO_2_) ratio (an indicator of respiratory efficiency) in mechanically ventilated patients. There is also a need for continuation of oral thromboprophylaxis, for example, with the usage of rivaroxaban, in ambulatory treatment, which reduces the risk of thromboembolic events during the first month after COVID-19 disease [92]. Of course, sustained anticoagulation management is highly recommended for patients suffering from AF, an undeniable risk factor of hypercoagulability and ischemic stroke [93].

## 7. Limitations

There is a need for conducting more long-term studies focused on detecting cases of NOAF connected with COVID-19 infection, including division of patients into age groups. Moreover, there are not enough studies concentrated on differentiation in terms of which variant of coronavirus is responsible for most of these cases. The current lack of extensive research is probably caused by the relatively short period of time (around six years) that has passed since the beginning of the pandemic.

Usage of telemonitoring and AI is also not free of limitations. The suggested methods of predicting the onset of COVID-19 infection are not specific for this disease—changes in HRV occur in different situations, which have an influence on the parasympathetic system and lead to systemic inflammation, including infectious diseases, alcohol, smoking, or the work environment. Additionally, even the most developed AI algorithms are not able to gather and correlate all of the patient’s data and cannot replace experienced doctor analysis or medical examination, such that neither physicians nor patients should only rely on the results shown by wearable devices. There are also differences in effectiveness of the types of ECG monitoring. Holter ECG is characterized by a short duration (24 h), which poses a risk of missing periodic episodes of arrhythmias. Usage of ECG patches demands implementation of digital analysis. PPG is not fully sensitive and specific, so it could generate inadequate notifications. Smartphone-based ECG may not detect asymptomatic episodes of AF because it requires activation by the patient who notices symptoms of arrhythmia, like palpitations or dyspnea.

## 8. Conclusions

AF is one of the most important cardiovascular complications associated with SARS-CoV-2 infection, resulting from a complex interplay of systemic inflammation, myocardial injury, endothelial dysfunction, and autonomic imbalance [15,20,23]. Both pre-existing AF and NOAF are linked to severe COVID-19 and worse in-hospital outcomes, including higher rates of ICU admission, mechanical ventilation, thromboembolic events, and mortality [1,3,39,42].

In COVID-19, AF represents a marker of global physiological stress and multi-organ dysfunction rather than an independent pathogenic process [39,41]. Cytokine storm, RAAS activation, and downregulation of ACE2 promote myocardial fibrosis and electrical remodeling [15,20,24], while hypoxia, metabolic disturbances, and sympathetic overactivation further enhance arrhythmogenesis [33,34].

NOAF significantly increases short-term mortality risk, whereas persistent AF carries the highest long-term risk [41,45]. After COVID-19, many patients remain vulnerable to AF and other rhythm disturbances due to lingering myocardial inflammation and structural remodeling [46,47], with elevated risk lasting at least one year [4,43].

For this reason, long-term rhythm monitoring is essential. Telemedicine and digital tools—ECG patches, ILRs, MCOT, and PPG-based smartwatches—allow for early detection of subclinical AF [71,79,80]. AI algorithms, including CNNs, LSTMs, and hybrid models, improve diagnostic accuracy and risk stratification [83,84]; for example, PMcardio AI achieves accuracy comparable to cardiologists [9].

Post-COVID care should focus on early arrhythmia detection, optimization of anticoagulation, and management of cardiovascular risk factors. Telecardiology can reduce rehospitalizations and improve long-term outcomes [6,51,78]. Large registries of COVID-19 survivors are also needed [79,81].

Although vaccination does not directly affect AF incidence, it significantly reduces the risk of severe infection and cardiovascular complications [86,87]. Continued research on vaccine safety, viral pathophysiology, and long-term cardiac effects is essential [88,89].

In summary, AF in COVID-19 is both a prognostic marker and a therapeutic target. Combining traditional cardiology with telemedicine and AI-based monitoring can reduce complications and improve quality of life in the post-pandemic era.

## Figures and Tables

**Table 2:** Comparison of methods of patient ECG monitoring [79,80].

Method	Type of Record	Typical Monitoring Duration	Main Advantages	Main Limitations	Typical Usage After COVID-19
24 h Holter ECG ^2^	Continuous three-lead/two-lead ECG	24 h	Widely available, inexpensive	Short monitoring duration—it is easy to miss intermittent episodes of arrhythmias	First-line test for patients with symptoms persisting for a short time after hospitalization
ECG patch	Continuous single-lead ECG	72 h–14 d	Longer recording, better patient tolerance	More expensive; obligatory digital analysis	Ambulatory detection of intermittent episodes of AF ^1^
Smartwatch (PPG ^4^ + 1-lead ECG)	Continuous/intermittent PPG + on-demand single-lead ECG	Continuous/passive alerts	Highly scalable, ability of passive long-term detection	PPG may generate false notifications; required ECG confirmation; differences in quality between devices	Screening and long-term remote monitoring of convalescents
ILR ^3^	Continuous subcutaneous ECG	Years	Detects most events; effective in long-term monitoring	Invasive, expensive	Monitoring of patients with high risk of cardiovascular incidents (for example, after ischemic stroke)
Event-/patient-activated ECG (smartphone-based)	Short single-lead ECG, patient-activated	Days–weeks	Good for symptomatic episodes; encourages patients to cooperate with doctors; low cost	Missing asymptomatic incidents	Registering recurrent episodes in patients who are willing to report their symptoms

^1^ Atrial fibrillation, ^2^ electrocardiogram, ^3^ implantable loop recorder, ^4^ photoplethysmogram.

## Data Availability

No new data were created or analyzed in this study.

## Abbreviations

The following abbreviations are used in this manuscript:

aHR	Adjusted Hazard Ratio
aOR	Adjusted Odds Ratio
ABP	Ambulatory Blood Pressure
ACE2	Angiotensin-Converting Enzyme 2
AAD	Antiarrhythmic Drug
AF	Atrial Fibrillation
AFL	Atrial Flutter
AI	Artificial Intelligence
AMI	Acute Myocardial Infarction
Ang2	Angiopoietin-2
ARDS	Acute Respiratory Distress Syndrome
ATII	Angiotensin II
ATP	Adenosine Triphosphate
BMI	Body Mass Index
BNP	B-type Natriuretic Peptide
BPM	Beats Per Minute
CAC	Coronary Artery Calcium
CAD	Coronary Artery Disease
CaMKII	Calcium/Calmodulin-Dependent Protein Kinase II
CIED	Cardiac Implantable Electronic Device
COPD	Chronic Obstructive Pulmonary Disease
COVID	Coronavirus Disease 2019
CNN	Convolutional Neural Network
CRP	C-Reactive Protein
CT	Computed Tomography
DCNN	Deep Convolutional Neural Network
DOAC	Direct Oral Anticoagulant
DVT	Deep Vein Thrombosis
EAT	Epicardial Adipose Tissue
ECG	Electrocardiogram
ECMO	Extracorporeal Membrane Oxygenation
EHRs	Electronic Health Records
FiO_2_	Fraction of Inspired Oxygen
GRU	Gated Recurrent Unit
HIF	Hypoxia-Inducible Factor
HMM	Hidden Markov Model
HRT	Heart Rate Turbulence
HRV	Heart Rate Variability
ICU	Intensive Care Units
IK1	Inward Rectifier Potassium Current (type 1)
IL	Interleukin
ILR	Implantable Loop Recorder
INR	International Normalized Ratio
KDR	Kinase Insert Domain Receptor
LMWH	Low-Molecular-Weight Heparin
LSTM	Long Short-Term Memory
mRNA	Messenger Ribonucleic Acid
MACE	Major Adverse Cardiovascular Events
MCOT	Mobile Cardiac Outpatient Telemetry
MMP	Matrix Metalloproteinase
NAD^+^	Nicotinamide Adenine Dinucleotide
NOAF	New-Onset Atrial Fibrillation
PaO_2_	Partial Pressure of Oxygen
PE	Pulmonary Embolism
PIMS-TS	Pediatric Inflammatory Multisystem Syndrome Temporally Associated with SARS-CoV-2
PPG	Photoplethysmogram
RAAS	Renin–Angiotensin–Aldosterone System
RhoA	Ras Homolog Family Member A
RHR	Resting Heart Rate
RNN	Recurrent Neural Network
RR	Respiratory Rate
RRT	Renal Replacement Therapy
RT-PCR	Reverse-Transcriptase Polymerase Chain Reaction
RyR	Ryanodine Receptor
SARS-CoV-2	Severe Acute Respiratory Syndrome Coronavirus 2
STE	Speckle Tracking Echocardiography
TEE	Transesophageal Echocardiography
TTE	Transthoracic Echocardiography
TGF-β1	Transforming Growth Factor Beta 1
TNF-α	Tumor Necrosis Factor Alpha
UFH	Unfractionated Heparin
VEGF	Vascular Endothelial Growth Factor
VKA	Vitamin K Antagonist
